# A Collection of Plates Illustrating the Theory and Practice of Midwifery; Accompanied by an Explanatory Text

**Published:** 1842-10

**Authors:** 


Art. XIV.—*A Collection of Plates illustrating the Theory and Practice of Midwifery; accompanied by an Explanatory Text.* Translated from the French of Em. Debout, m.d.—*London*, 1841. Folio.

We notice this work merely on account of its demerits. The plates which illustrate it, though stated to be "designed by the author himself with great care," are with few exceptions unacknowledged copies from Hunter, Smellie, or other well-known originals. They are very indifferently executed, and the text which accompanies them is evidently the production of some ignorant compiler. Thus, for instance, the inclination of the pelvis is stated to be 45° instead of 60°, mollities ossium is spoken of as a peculiar disease described by Burns, and a direct communication is asserted to exist between the maternal and fœtal vessels in the placenta. The translation has been executed by some foreigner but ill acquainted with our language, and the book contains many typographical errors.

